# Supplementary material for: Allosteric inhibition of the epidermal growth factor receptor through disruption of transmembrane interactions
Source: J Biol Chem. 2023 Jun 12;299(7):104914. doi: 10.1016/j.jbc.2023.104914 (PMC10362150; doi:10.1016/j.jbc.2023.104914)

**Supporting Information**


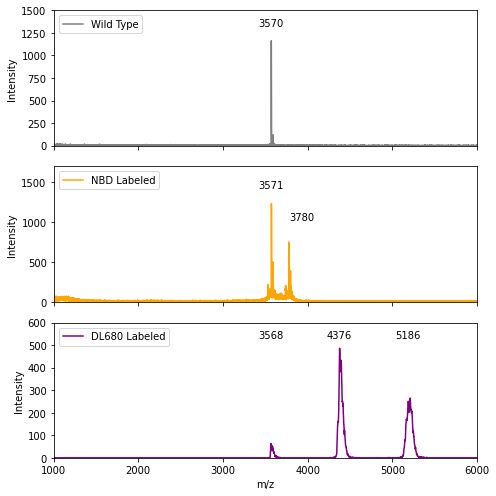


**Supplementary Figure 1: MALDI-TOF of PET1, PET1-NBD, and PET1 DL680.**

**
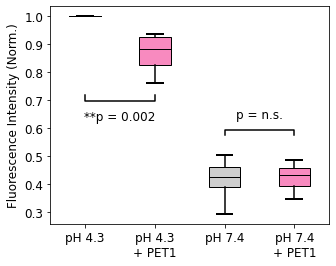
**

**Supplemental Figure 2: PET1 decreases the fluorescence intensity of TM-EGFR only at acidic pH.** The fluorescence spectra of TM-EGFR in POPC lipid vesicles at pH 4.3 and 7.4 in the presence (pink) or absence (grey) of PET1. Box plot conveys the fluorescence at the max of the curve. N = 6 with each biological replicate normalized to pH 4.3 conditions. Statistical analysis was performed using a Kruskal Wallis test -H(3) = 19.75, *p* = 0.0002- with a Mann Whitney U test for comparisons between groups.


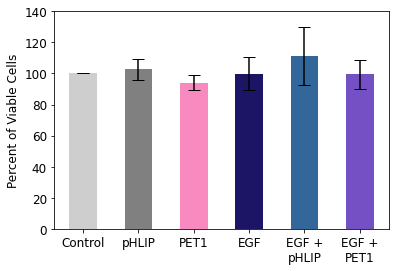


**Supplementary Figure 3: PET1 is not toxic to A375 cells.** MTS assay was performed to evaluate cell viability. The pHLIP peptide was used as a control for a pH responsive peptide with no toxicity (16). Within each biological replicate the number of untreated cells was normalized to 100% viability. N = 3. Error bars denote standard deviation of the mean.


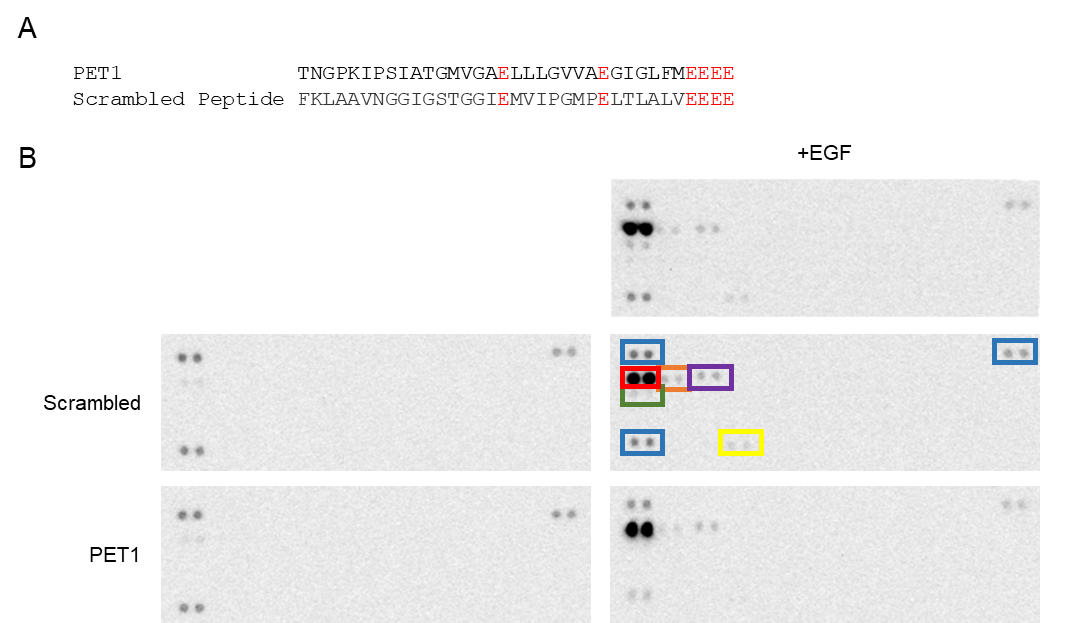


**Supplementary Figure 4: PET1 does not activate other RTKs. A,** Sequences of PET1 and the scrambled peptide (SP) used as a control. E residues were kept constant and are highlighted in red. **B,** Lysates of A431 cells were treated with or without PET1 overnight followed by a 5-minute treatment with or without EGF. Tyrosine phosphorylation on a variety of RTKs was probed with a Human Phospho-RTK Array Kit. Three sets of reference dots (blue) allow determination of RTK identities. Specific RTK are boxed: EGFR (red), ErbB2 (orange), ErbB3 (purple), MerTK (green), and EphB2 (yellow). Other RTKs did not show phosphorylation in any conditions: ALK/CD246, Axl, DDR1, DDR2, Dtk, EphA1, EphA2, EphA3, EphA4, EphA5, EphA6, EphA7, EphA10, EphB1, EphB3, EphB4, ErbB2, ErbB3, ErbB4, ErbB6, FGFR1, FGFR2 alpha, FGFR3, FGFR4, Flt-3/Flk, HGF R/c-MET, IGF-I R, Insulin R/CD220, M-CSF R, Mer, MSP R/Ron, MuSK, PDGF R alpha, Ret, ROR1, ROR2, Ryk, PDGFRβ, SCF R/c-kit, Tie-1, Tie-2, TrkA, TrkB, TrkC, VEGF R1/Flt-1, VEGF R2/KDR, and VEGF R3/Flt-4. Blot identities can be found at https://resources.rndsystems.com/pdfs/datasheets/ary001b.pdf?v=20220613&_ga=2.236641154.205998430.1655143431-478466794.1651682163

**Supplementary Figure 5.** 2D Plots showing the configurational transition of EGFR TM dimers over the simulation time considering the inter-helical angle vs inter-helical distance for the EGFR TM-only (**A**) and EGFR-PET1 (**B**). Results from all 4 trajectories are shown. The plots are colored based on the inter-helical distance from 0.5-1.0 nm (green), 1-1.5 nm (red) and 1.5-2.0 nm (purple).


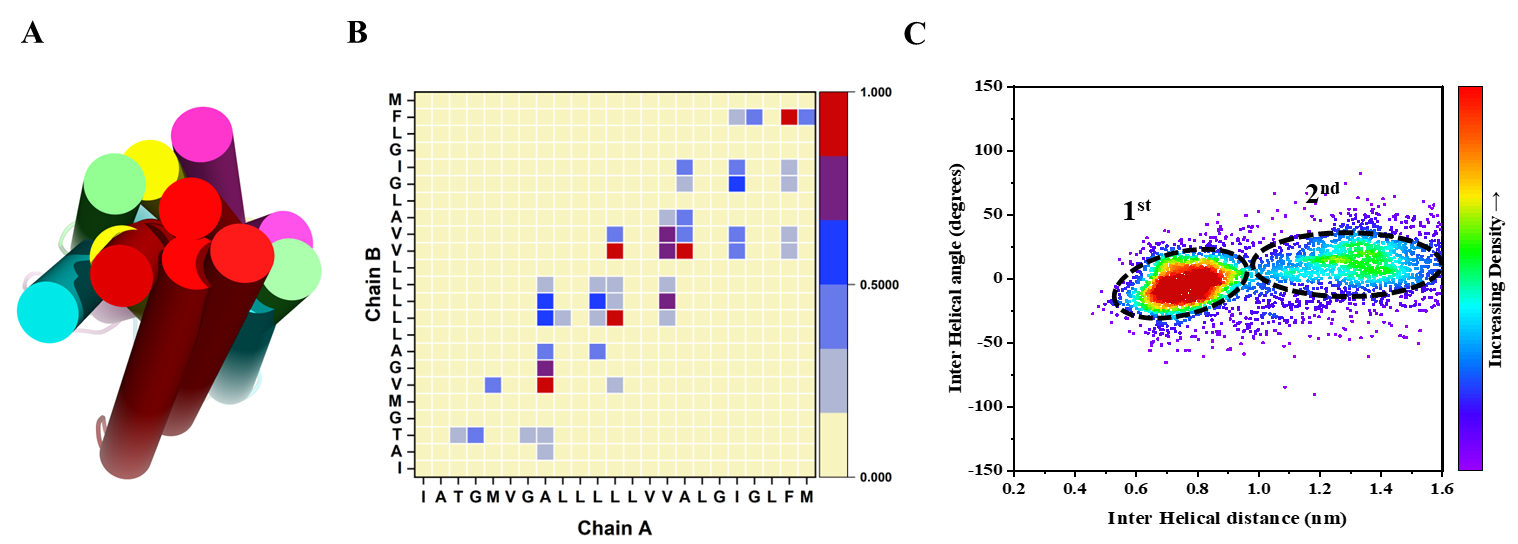

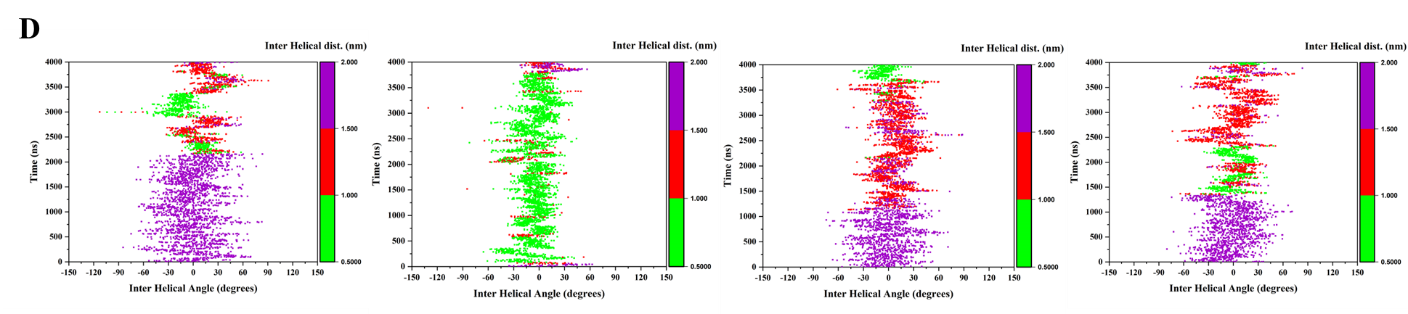


**Supplementary Figure 6.** Association of the EGFR TMs regions in the presence of the Scrambled peptide (SP). **A,** Superimposition of the central conformers for all the four CG simulations in case of EGFR-SP systems. The SP peptide is shown in red and the EGFR TMs in different colors. **B,** Contact map interface between the EGFR TMs for EGFR-SP systems. Data from the last 1 µs simulations are considered for all the 4 simulations. Contact maps are calculated with a cut off 5 Å. The color scale (white to blue to red) indicates the fractional occupation of TM contacts (0 to 1). **C,** 2D distribution plot (interhelix angle vs. distance) between the EGFR TMs for EGFR-SP systems. Populated clusters are named as 1^st^, 2^nd^ and 3^rd^. Data from the last 1 µs simulations are considered for all the 4 simulations. **D,** Plots showing the configurational transition of EGFR TM dimers over the simulation time considering the inter-helical angle vs inter-helical distance.


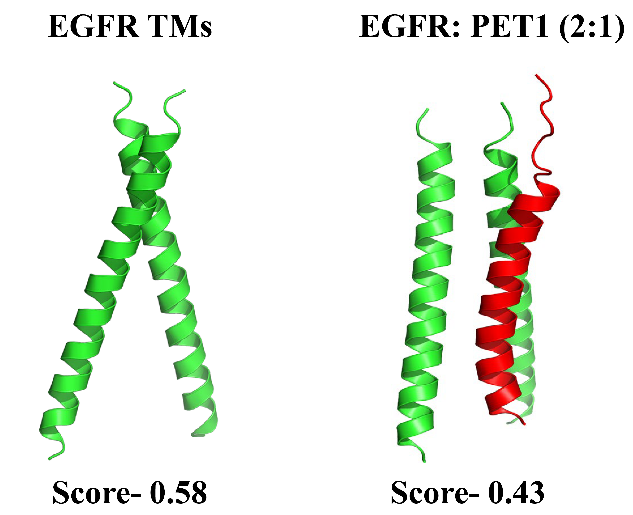


**Supplementary Figure 7.** Comparison of AlphaFold-Multimer predicted structures of EGFR TM (green) homodimer and in complex with PET1 (red). We also predicted the structure of the EGFR: SP (2:1), but the AlphaFold-Multimer score was low (0.3), probably because since the SP sequence was randomly arranged in the SP, there are few homologous sequences available in the training dataset. Therefore, the SP prediction was poor and accuracy of that prediction is uncertain, and it is not shown.

**Table S1:** Comparison of CG simulations of EGFR TM-only with EGFR with PET1 and with PET1 scrambled peptide (SP). The last column compares with the NMR EGFR structure. Central conformers of the top 4 clusters for the combined simulations are considered for the comparison using the PREDDIMER method where it compares the Fscor and the crossing angle between the TM helices. Fscor values > 2.5 were considered stable TM dimer structure. In presence of the PET1 peptide, the association of the EGFR TMs are weaker with the Fscor value < 2.5 (shown in red) for the two most populated clusters. PREDDIMER does not calculate the crossing angle if the Fscor is ‘0’ which are the cases for some of the EGFR-PET1 conformers.


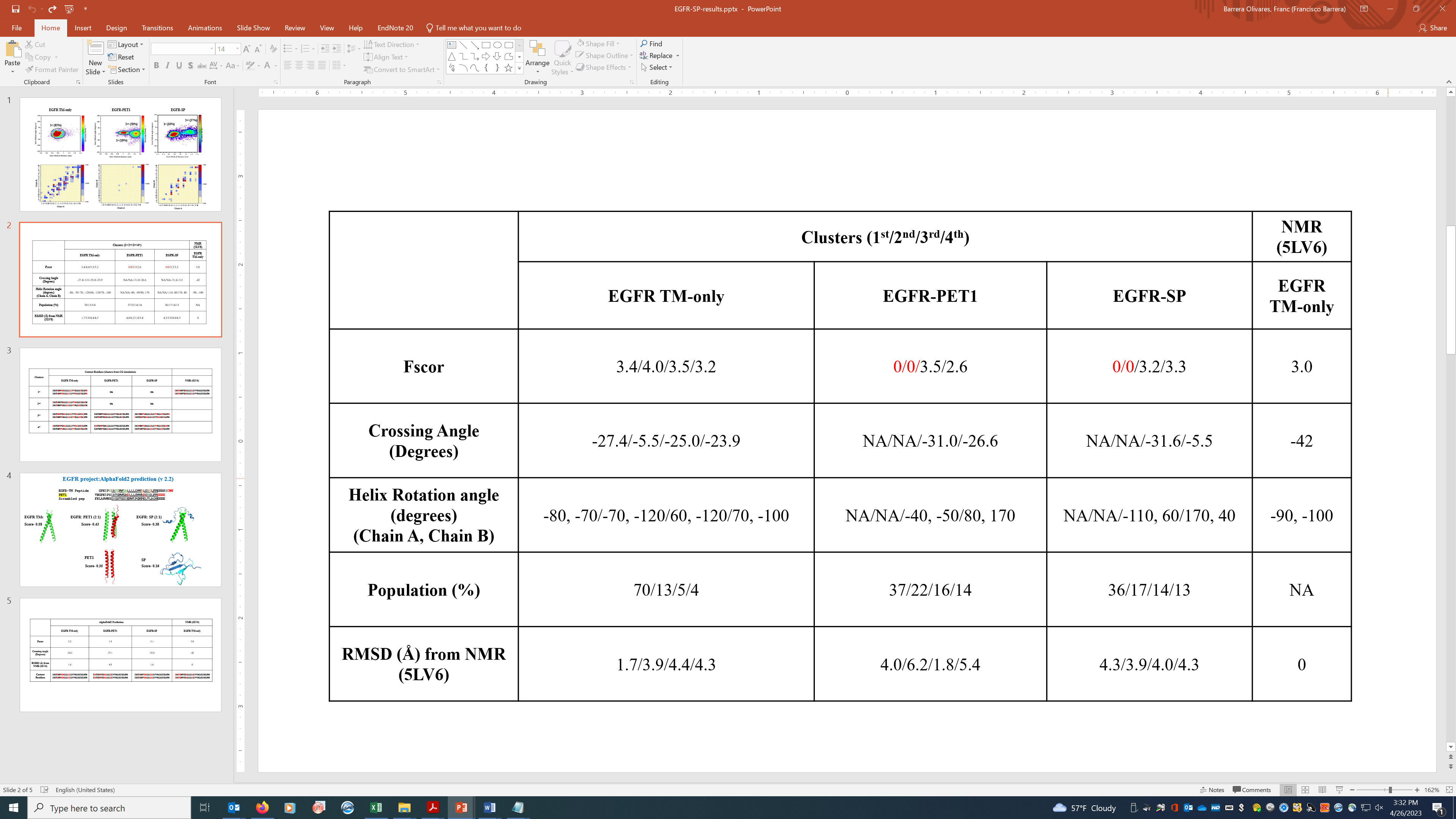

Supplement: Supporting Figures S1–S7 and Tables S1 [file mmc1.docx]
